# Study on the influencing factors of the first-line treatment response to primary immune thrombocytopenia in children

**DOI:** 10.3389/fped.2025.1679463

**Published:** 2025-10-03

**Authors:** Bihan Zhang, Qian Hu, Qiaodao Wu, Jianying Ning

**Affiliations:** Department of Pediatrics, The First Affiliated Hospital of Shihezi University, Shihezi, Xinjiang, China

**Keywords:** ITP in children, first-line treatment, therapeutic effect, plateletparameters, P-Glycoprotein

## Abstract

**Aims:**

This study aims to investigate the factors influencing the first-line treatment response in children with primary immune thrombocytopenia (ITP) and to evaluate the predictive value of these indicators for therapeutic outcomes.

**Methods:**

A total of 140 pediatric patients diagnosed with ITP at the Pediatrics Department of the First Affiliated Hospital of Shihezi University, between June 2022 and December 2024, were enrolled. Patients were grouped according to the type of first-line treatment and treatment response. Laboratory tests such as platelet parameters, P-glycoprotein (P-gp), T lymphocyte subsets, immunoglobulins, and complements were collected and analyzed.

**Results:**

Multivariate logistic regression analysis identified elevated P-gp and platelet-to-lymphocyte ratio (PLR), decreased of CD4^+^/CD8^+^ ratio and complement 3 (C3) levels as independent risk factors for glucocorticosteroid (GC) treatment failure in children with ITP. Receiver operating characteristic curve (ROC) analysis identified the areas under the curve (AUCs) were 0.772, 0.763, 0.731, and 0.731. The combined model yielded an AUC of 0.919. Elevated PLR and immunoglobulin G (IgG) were independent risk factors for intravenous immune globulin (IVIG) treatment failure.ROC analysis showed that PLR and IgG were predictive of IVIG treatment failure in children with ITP. The AUCs were 0.769 and 0.722, respectively. The combined model yielded an AUC of 0.810.

**Conclusions:**

Elevated P-gp and PLR, along with reduced CD4^+^/CD8^+^ ratio and C3 levels, are independent risk factors for GC treatment failure in children with ITP. For IVIG therapy, increased PLR and IgG levels are associated with poor response. These indicators demonstrate predictive value for first-line treatment efficacy, with combined marker analysis offering superior predictive accuracy compared to individual indicators.

## 1 Introduction

Primary immune thrombocytopenia (ITP) is a common autoimmune hemorrhagic disease in children. Epidemiological data from the United States report an overall incidence of 3.9 per 100,000 children, with a higher incidence in females (4.4/100,000) compared to males (3.4/100,000) (1). The prevalence of chronic ITP is estimated to be between 9.5 and 11.2 per 100,000 (1). In China, the annual incidence of pediatric ITP ranges from 1.6 to 5.3 per 100,000, with chronic cases accounting for approximately 0.8–1.25 per 100,000 (2, 3). Glucocorticoid (GC) and Intravenous immune globulin (IVIG) are the first-line treatments for children with ITP. While the majority of pediatric patients respond favorably to these therapies, approximately 5%–10% exhibit poor initial response, experience relapses, or progress to chronic or refractory ITP (2, 3). Despite extensive clinical use, the mechanisms underlying treatment resistance remain incompletely understood. Therefore, identifying reliable predictors of treatment response is crucial for optimizing therapeutic strategies, improving prognosis, and minimizing unnecessary side effects and healthcare costs.

Platelets play a central role in maintaining vascular integrity and facilitating hemostasis. Platelet parameters serve as important indicators of megakaryocyte activity, platelet activation, functional status, and lifespan in circulation. These parameters are closely associated with disease severity, progression, and treatment efficacy in ITP (4). The pathogenesis of ITP is multifactorial, involving both humoral and cellular immune dysregulation. Recent studies have highlighted the role of abnormal T cell-mediated immunity, particularly the imbalance in T lymphocyte subsets and cytokine profiles, in the development of chronic and refractory ITP (5). P-glycoprotein (P-gp), a transmembrane transport protein encoded by human multidrug resistance gene 1, transports drugs from intracellular to extracellular through active transport. Overexpression of P-gp reduces intracellular drug accumulation and diminishes therapeutic efficacy, contributing to treatment resistance in several diseases (6, 7). Additionally, immunoglobulin levels reflect the status of humoral immunity, while the complement system plays a critical role in immune regulation, including macrophage activation, classical pathway initiation, and membrane attack complex formation (8, 9).

Some studies suggest that the levels of the above factors are associated with disease severity and prognosis in ITP. Although numerous studies have attempted to identify biomarkers predictive of treatment response, conclusive evidence remains limited. In this study, we aim to investigate the predictive value of platelet indices, P-gp, T lymphocyte subsets, immunoglobulins, and complement levels in children receiving different first-line therapies. The goal is to provide early, objective indicators to guide individualized treatment strategies, reduce ineffective therapy and complications, and enhance understanding of ITP pathophysiology to inform future therapeutic approaches.

## 2 Methods

### 2.1 Study population

This study included 140 pediatric patients diagnosed with ITP who were the Department of Pediatrics, the First Affiliated Hospital of Shihezi University from June 2022 to December 2024. All cases were observed for 6 months to determine efficacy. Inclusion criteria were based on established diagnostic guidelines (2): (1) Platelet count <100 × 10^9^/L confirmed on at least two separate occasions, with no significant morphological abnormalities observed in peripheral blood smear microscopy; (2) Absence of splenomegaly on physical examination; (3) Bone marrow cytology tests indicating increased on megakaryocytes or normal megakaryocytes with maturation disorders. Exclusion criteria: (1) Secondary thrombocytopenia; (2) Use of medications known to affect platelet counts; (3) Patients with severe infection, other autoimmune diseases, or malignant tumors during the study period; (4) Those who refused to participate or withdrew from the study. Ethical approval for this study was obtained from the institutional ethics Review board (Ethics approval number: KJ2024-257-01).

### 2.2 Treatment options

For patients with platelet counts between 10 and 50 × 10^9^/L, active bleeding without emergency treatment, bleeding risk and parents' need for medication, GC treatment was agreed after bone marrow aspiration examination and fully communicated with parents. Treatment regimen: oral prednisone tablets daily: 1–2 mg/(kg·d), the maximum dose was 60 mg daily, used for 7–14 days when the condition was stable, and the drug was stopped. The total course of treatment was less than 6 weeks.

Patients with active bleeding or risk of bleeding, platelet counts below 20 × 10^9^/L, or those unable to undergo immediate bone marrow examination were treated with IVIG. The regimen included intravenous immunoglobulin at a dose of 0.8–1 g/(kg·d) for 1–2 days.

### 2.3 Efficacy and group assignment

Based on treatment regimen and therapeutic response, patients were divided into GC treatment group (GC effective group, GC ineffective group) and IVIG treatment group (IVIG effective group, IVIG ineffective group). According to the guidelines for the diagnosis and treatment of the disease, both complete response (no bleeding after treatment and platelet count ≥100 × 10^9^/L) and response (no bleeding after treatment, platelet count ≥30 × 10^9^/L and increase by two times or more than the baseline value) were included in the effective group (2). First-line treatment failure was defined as: failure to achieve treatment response at 1 week and 1 month evaluation after the application of first-line drugs,and failure to maintain treatment response (6 months) or the need for long-term use of glucocorticoids to maintain response (2).

### 2.4 Study methods

Clinical data of the children were collected: gender, age, Platelet count (PLT), mean platelet volume (MPV), Plateletcrit (PCT), Platelet volume distribution width (PDW), Lymphocyte count (Lymphocyte count, LYMPH), Platelet-to-lymphocyte ratio (platelet-to-lymphocyte ratio, PLR), P-glycoprotein, percentage of CD4^+^T lymphocytes, percentage of CD8^+^T lymphocytes, CD4^+^/CD8^+^ ratio, Immunoglobulin G(IgG), Immunoglobulin A(IgA), Immunoglobulin M(IgM), Complement 3 (C3) and Complement 4 (C4).

### 2.5 Statistical analysis

SPSS 27.0 statistical software was used for data analysis. Categorical variables were presented as counts (n) and percentages (%), and the differences between groups were compared using the chi-square test or Fisher exact probability method. Normal distribution measurement data were expressed as $\left(\bar{x}\pm s\right)$, and the differences between groups were compared by independent sample *t* test. Non-normal distribution data were expressed as median (interquartile range), namely M(P25, P75), and non-parametric test was used for comparison between groups. The factors with statistically significant differences in the above inter-group comparisons were included in the binary Logistic regression analysis to identify independent risk factors and evaluate their impacts. The Receiver operating characteristic curve (ROC) was used to calculate the best diagnostic cut-off value and the Area under curve (AUC). *P* < 0.05 was considered statistically significant.

## 3 Results

### 3.1 GC treatment group

#### 3.1.1 Comparison of clinical data between GC treatment groups

Among the 74 children in the GC treatment group, 58 were classified as GC responders and 16 as non-responders. The percentage of PLR, P-gp and CD8^+^T lymphocytes in the GC effective group were lower than those in the ineffective group (*Z* = −3.309, −3.204, −2.252, all *P* < 0.05). Conversely, the levels of LYMPH, CD4^+^/CD8^+^ ratio and C3 in GC effective group were higher than those in GC ineffective group (*Z* = −3.086, *t* = 2.916, 2.877, all *P* < 0.05). There was no significant difference in other indicators between the two groups (*P* > 0.05) (Table 1).

**Table 1:** Comparison of clinical data in the GC treatment group.

Variables	GC effective group (*n* = 58)	GC ineffective group (*n* = 16)	*t*/*Z*/*χ*^2^	*P*-value
Gender [*n* (%)]				
Male	32 (55.17)	7 (43.75)	0.656	0.418
Female	26 (44.83)	9 (56.25)		
Age (years)	4.21 (2.08, 7.21)	4.42 (2.42, 7.19)	−0.440	0.660
Bleeding score				
0	42 (72.41)	14 (87.50)	–	0.327^a^
1	16 (27.58)	2 (12.50)		
Therapeutic dose	33.00 (26.00, 46.00)	34.00 (25.25, 45.75)	−0.211	0.833
PLT (×10^9^/L)	28.57 ± 7.74	30.56 ± 9.51	−0.867	0.389
MPV (fl)	10.10 (9.40, 12.00)	10.70 (9.83, 12.10)	−0.992	0.321
PCT (%)	0.030 ± 0.010	0.033 ± 0.009	−0.943	0.349
PDW (%)	15.80 (15.20, 16.30)	16.15 (14.40, 17.30)	−0.816	0.415
LYMPH (×10^9^/L)	2.03 (1.64, 2.59)	1.47 (1.24, 2.10)	−3.086	0.002
PLR (%)	12.62 (10.15, 16.84)	19.08 (13.81, 26.65)	−3.309	0.001
P-gp (ng/ml)	2.80 (2.12, 3.14)	3.26 (3.00, 3.97)	−3.204	0.001
CD4^+^T (%)	33.19 (26.99, 39.85)	31.32 (27.61, 34.60)	−0.992	0.321
CD8^+^T (%)	33.09 (29.65, 37.75)	35.49 (34.56, 40.15)	−2.252	0.024
CD4^+^/CD8^+^	1.02 ± 0.22	0.85 ± 0.15	2.916	0.005
Immunoglobulins and complements (g/L)				
IgG	10.05 (7.90, 20.00)	14.74 (10.65, 18.42)	−1.104	0.269
IgA	1.11 (0.75, 1.89)	1.01 (0.53, 1.21)	−1.209	0.227
IgM	0.91 (0.74, 1.21)	1.03 (0.77, 1.60)	−1.098	0.272
C3	0.92 ± 0.29	0.70 ± 0.23	2.877	0.005
C4	0.18 (0.16, 0.22)	0.18 (0.16, 0.19)	−0.659	0.510

^a^ Fisher's exact test.

GC, glucocorticosteroid; PLT, platelet count; MPV, mean platelet volume; PCT, plateletcrit; PDW, platelet distribution width; LYMPH, lymphocyte count; PLR, platelet-to-lymphocyte ratio; P-gp, P-glycoprotein; IgG, immunoglobulin G; IgA, immunoglobulin A; IgM, immunoglobulin M; C3, complement 3; C4, complement 4.

#### 3.1.2 Logistic regression analysis of factors influencing the efficacy of GC in the GC therapy group

Logistic regression analysis showed that the increase of PLR and P-gp, the decrease of CD4^+^/CD8^+^ ratio and C3 level were independent risk factors for ineffective GC treatment in children with ITP (*OR* = 1.13, *95%CI*: 1.01–1.26; *OR* = 4.80, *95%CI*: 1.46–15.79; *OR* = 0.01, *95%CI*: 0.00–0.32; *OR* = 0.01, *95%CI*: 0.00–0.36, all *P* < 0.05) (Table 2).

**Table 2:** Logistic regression analysis of factors influencing the efficacy of GC in the GC therapy group.

Variables	*B*	*S.E.*	*Wald*	*P*-value	*OR*	*95%CI*
PLR	0.12	0.06	4.67	0.031	1.13	1.01–1.26
P-gp	1.57	0.61	6.66	0.010	4.80	1.46–15.79
CD4^+^/CD8^+^	−5.71	2.33	5.99	0.014	0.01	0.00–0.32
C3	−4.57	1.82	6.34	0.012	0.01	0.00–0.36

B, unstandardized regression coefficient; S.E., standard error; Wald, wald statistic; OR, odds ratio; CI, confidence interval; PLR, platelet-to-lymphocyte ratio; P-gp, P-glycoprotein; C3, complement 3.

#### 3.1.3 The predictive value of factors for the efficacy of GC in children with ITP

**Figure 1:**
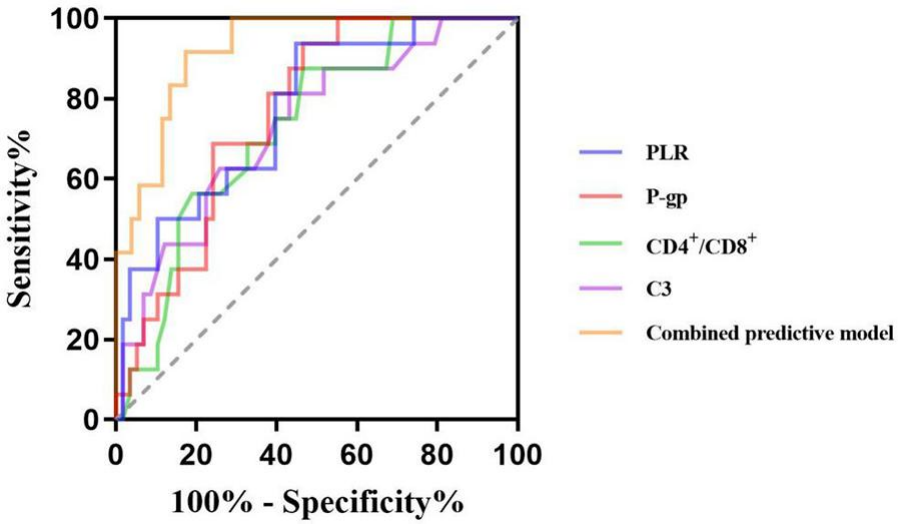
Predictive value of PLR, P-gp, CD4+/CD8+ ratio, and C3 for inefficacy of GC therapy in pediatric ITP patients.

The ROC curve showed that the cut-off values of PLR, P-gp, CD4^+^/CD8^+^ ratio and C3 for predicting ineffective GC treatment in pediatric ITP patients were 12.85%, 2.83 ng/ml, 1.02 and 0.86 g/L, respectively, and the *AUC* were 0.772, 0.763, 0.731 and 0.731, respectively. The *95%CI* were 0.65–0.90, 0.65–0.87, 0.60–0.86 and 0.60–0.87, the sensitivity were 93.8%, 93.8%, 53.4% and 56.9%, and the specificity were 65.2%, 63.4%, 87.5% and 81.2%. The *AUC* of the combined prediction of the four items was 0.919, *95%CI* was 0.86–0.98, and the sensitivity and specificity were 87.5% and 81.8%, respectively (Table 3, Figure 1).

**Table 3:** Predictive value of PLR, P-gp, CD4^+^/CD8^+^ ratio, and C3 for inefficacy of GC therapy in pediatric ITP patients.

Index	*AUC*	Sensitivity (%)	Specificity (%)	Youden index	Cut off value	*95%CI*
PLR (%)	0.772	93.8	65.2	0.490	12.85	0.65–0.90
P-gp (ng/ml)	0.763	93.8	63.4	0.472	2.83	0.65–0.87
CD4^+^/CD8^+^	0.731	53.4	87.5	0.409	1.02	0.60–0.86
C3 (g/L)	0.731	56.9	81.2	0.381	0.86	0.60–0.87
Combined predictive model	0.919	87.5	81.8	0.703	–	0.86–0.98

AUC, area under curve; CI, confidence interval; PLR, platelet-to-lymphocyte ratio; P-gp, P-glycoprotein; C3, complement 3.

### 3.2 IVIG treatment group

#### 3.2.1 Comparison of clinical data between IVIG treatment groups

Among the 66 children in the IVIG treatment group, 55 cases were in the effective IVIG group, and 11 cases were in the ineffective IVIG group. The levels of PLR and IgG in the effective IVIG group were lower than those in the ineffective group (*Z* = −2.796, −2.316, both *P* < 0.05). The levels of serum LYMPH and IgM were higher than those in the ineffective group (*Z* = −4.054, −2.075, both *P* < 0.05). There was no statistically significant difference in other indicators between the groups (*P* > 0.05) (Table 4).

**Table 4:** Comparison of clinical data in the IVIG treatment group.

Variables	IVIG effective group (*n* = 55)	IVIG ineffective group (*n* = 11)	*t*/*Z*/*χ*^2^	*P*-value
Gender [*n* (%)]				
Male	24 (43.64)	4 (36.36)	–	0.747^a^
Female	31 (56.36)	7 (63.64)		
Age (years)	4.17 (2.25, 7.42)	5.50 (1.67, 8.08)	−0.327	0.744
Bleeding score				
0	27 (49.09)	4 (36.36)	–	0.457^a^
1	21 (38.18)	4 (36.36)		
2	3 (5.46)	2 (18.19)		
3	4 (7.27)	1 (9.09)		
Therapeutic dose	17.00 (13.00, 25.00)	19.00 (11.50, 27.00)	−0.207	0.836
PLT (×10^9^/L)	8.00 (5.00, 14.00)	11.00 (6.00, 17.00)	−1.095	0.273
MPV (fl)	11.05 ± 1.75	10.54 ± 2.05	0.856	0.395
PCT (%)	0.008 (0.005, 0.018)	0.012 (0.005, 0.018)	−0.793	0.428
PDW (%)	15.80 (15.20, 16.30)	16.20 (15.20, 16.70)	−1.034	0.301
LYMPH (×10^9^/L)	2.30 (1.91, 2.79)	1.30 (1.03, 2.03)	−4.054	<0.001
PLR (%)	3.61 (1.86, 6.52)	9.36 (4.44, 12.30)	−2.796	0.005
P-gp (ng/ml)	2.63 (2.0, 3.20)	3.09 (2.37, 3.44)	−1.342	0.179
CD4^+^T (%)	31.66 ± 7.42	27.23 ± 6.85	1.830	0.072
CD8^+^T (%)	29.13 ± 6.45	27.54 ± 7.91	0.716	0.477
CD4^+^/CD8^+^	1.06 (0.88, 1.46)	1.05 (0.61, 1.50)	−0.353	0.724
Immunoglobulins and complements (g/L)				
IgG	13.10 (8.87, 18.30)	18.25 (15.20, 22.40)	−2.316	0.021
IgA	1.13 (0.75, 1.87)	0.98 (0.54, 1.43)	−0.853	0.394
IgM	1.07 (0.81, 1.73)	0.79 (0.57, 1.19)	−2.075	0.038
C3	0.91 ± 0.24	0.82 ± 0.21	1.177	0.244
C4	0.19 ± 0.08	0.16 ± 0.08	0.926	0.358

^a^ Fisher's exact test.

IVIG, intravenous immune globulin; PLT, platelet count; MPV, mean platelet volume; PCT, plateletcrit; PDW, platelet distribution width; LYMPH, lymphocyte count; PLR, platelet-to-lymphocyte ratio; P-gp, P-glycoprotein; IgG, immunoglobulin G; IgA, immunoglobulin A; IgM, immunoglobulin M; C3, complement 3; C4, complement 4.

#### 3.2.2 Logistic regression analysis of factors influencing the efficacy of IVIG in the IVIG therapy group

Logistic regression analysis showed that PLR and IgG levels were independent risk factors for ineffective IVIG treatment in ITP children (*OR* = 1.27, *95%CI*: 1.05–1.53; *OR* = 1.16, *95%CI*: 1.01–1.34, all *P* < 0.05) (Table 5).

**Table 5:** Predictive value of PLR and IgG levels for inefficacy of IVIG therapy in ITP patients.

Variables	*B*	*S.E.*	*Wald*	*P*-value	*OR*	*95%CI*	
PLR	0.24	0.10	6.26	0.012	1.27	1.05	1.53
IgG	0.15	0.07	4.64	0.031	1.16	1.01	1.34
IgM	−1.56	0.88	3.18	0.075	0.21	0.04	1.17

B, unstandardized regression coefficient; S.E., standard error; Wald, wald statistic; OR, odds ratio; CI, confidence interval; PLR, platelet-to-lymphocyte ratio; IgG, immunoglobulin G; IgM, immunoglobulin M.

#### 3.2.3 The predictive value of factors for the efficacy of IVIG in children with ITP

**Figure 2:**
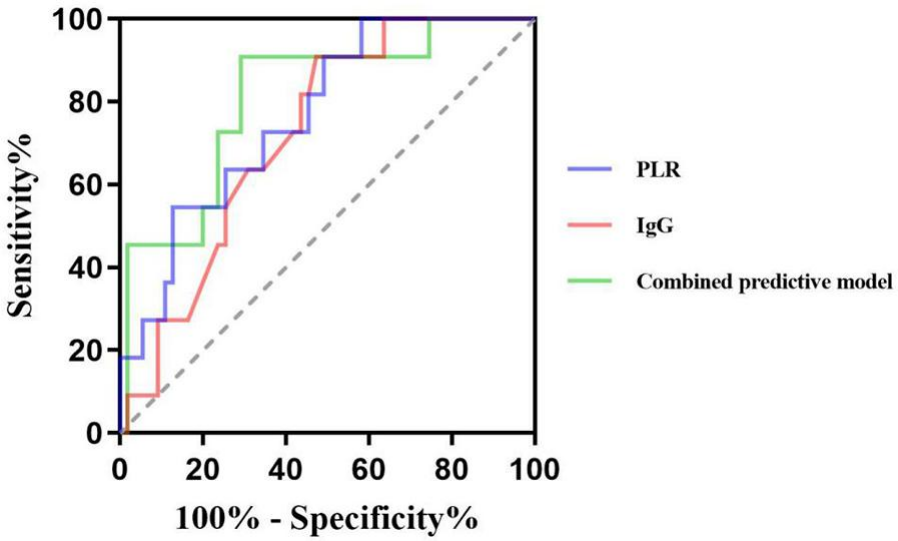
Predictive value of PLR and IgG for inefficacy of IVIG therapy in pediatric ITP patients.

The ROC curve showed that the cut-off values of PLR and IgG for predicting ineffective IVIG treatment in children with ITP were 8.91% and 13.84 g/L, respectively, and the *AUC* were 0.769 and 0.722, respectively, 95%CI were 0.63–0.91 and 0.59–0.86, respectively. The sensitivity and specificity were 54.5% and 90.9%, 87.3% and 52.7%, respectively. The *AUC* of the combined prediction of the two parameters was 0.810, *95%CI* was 0.67–0.95, and the sensitivity and specificity were 90.9% and 70.9%, respectively (Table 6, Figure 2).

**Table 6:** Predictive value of PLR and IgG for inefficacy of IVIG therapy in pediatric ITP patients.

Index	*AUC*	Sensitivity (%)	Specificity (%)	Youden index	Cut off value	*95%CI*
PLR (%)	0.769	54.5	87.3	0.418	8.91	0.63–0.91
IgG (g/L)	0.722	90.9	52.7	0.436	13.84	0.59–0.86
Combined predictive model	0.810	90.9	70.9	0.618	–	0.67–0.95

AUC, area under curve; CI, confidence interval; PLR, platelet-to-lymphocyte ratio; IgG, immunoglobulin G; IgM, immunoglobulin M.

## 4 Discussion

ITP is a prevalent hematological disease in children, typically presenting with an acute and self-limiting course. However, in clinical practice, there are still some patients who show no response to first-line drug treatment and progress to chronic or refractory outcomes. Many have explored potential biomarkers for evaluating therapeutic response in ITP, including megakaryocyte classification and quantity, ferritin level, IGFBP2 and BCL2 levels (10–12). However, the predictive effects of the above novel biomarkers have not yet been confirmed. In the absence of reliable prognostic tools, many patients undergo prolonged and repeated medication cycles, increasing the risk of drug-related adverse effects and ultimately requiring transition to second-line therapies. Although there are no relevant reports on the exact indicators for predicting the efficacy of first-line drugs for ITP at present, some literature suggests that in some patients with tumors and autoimmune diseases, platelet parameters, P-gp, T lymphocyte subsets, immunoglobulins and complements, etc. are related to the efficacy and prognosis of the disease (8, 9, 13–16).

Platelet parameters are key indicators of platelet quantity and function, closely associated with progression and outcome in ITP (13, 14). LYMPH and PLR can reflect the inflammatory level and disease activity of autoimmune diseases (17). Decreased LYMPH was a risk factor for ITP patients to progress to chronic and persistent conditions (18). Wang's study showed a significant correlation between reduced PLR and hormone resistance (19). Consistent with these findings, our study showed higher LYMPH and lower PLR in the GC effective group than in the ineffective group. The anti-platelet antibodies produced by humoral immunity damage the surface molecules of lymphocytes. The immune system's tolerance to its own antigens decreases, and it is unable to effectively suppress the autoimmune response, resulting in the exhaustion of lymphocytes. Yang's study showed that the AUC of PLR for predicting the prognosis of ITP in children was 0.680 (20). Our study showed PLR was an independent risk factor for GC ineffective treatment, with a cut-off value of 12.85%, AUC of 0.772, sensitivity of 93.8%, and specificity of 65.2%, indicating high sensitivity but moderate specificity.

P-gp mediates the cellular efflux of exogenous substances and metabolites, prevents harmful substances from being absorbed, and has a broad-spectrum drug transport function, but also contributing to multidrug resistance with individual variation (21, 22). In autoimmune diseases, elevated P-gp expression and activity have been observed in patients who are resistant or non-responsive to GC therapy, suggesting a correlation between P-gp levels and treatment efficacy (7, 23). While previous studies have focused on comparing the difference of P-gp expression between ITP patients and healthy controls or ITP patients with varying disease severity, few have examined its relationship with treatment outcomes (24). In this study, the level of P-gp in the GC effective group was lower than in the ineffective group, consistent with Guo's study that “P-gp expression level can affect the effect of glucocorticoid treatment” (15). The high level of P-gp expression in the ineffective group accelerates GC efflux, prevents the accumulation of hormones in the cell and hinders the binding process, reduces the pharmacological effect of hormones, affecting the curative effect and prognosis. Logistic regression analysis showed that P-gp was an independent risk factor for ineffective GC, consistent with Li's conclusion (25). ROC analysis showed the sensitivity and specificity were 93.8% and 63.4%, the cut-off value was 2.83 ng/ml, and the AUC was 0.763.

In both neoplastic and immune diseases, T lymphocyte subsets fluctuate with disease activity (26). In this study, the GC ineffective group exhibited a higher percentage of CD8^+^ T lymphocytes and a lower CD4^+^/CD8^+^ ratio compared to the effective group. This shift reflects an increase in cytotoxic (CD8^+^) T cells, which can directly induce platelet apoptosis and destruction, while the relative reduction in helper (CD4^+^) T cells weakens immune regulation, exacerbating cellular immune dysfunction (27, 28). GC inhibits the activation and proliferation of B lymphocytes, reduces the production of antiplatelet antibodies, blocks the formation of antigen-antibody complexes, and delays the phagocytosis of mononuclear macrophages (29). It has a good therapeutic effect on children with ITP dominated by humoral immune disorders, but has a poor therapeutic effect on children mediated by cellular immunity mainly characterized by high expression of CD8^+^T lymphocytes (29). The reason was analyzed to be the limited ability of GC to inhibit the cytotoxic function of CD8^+^T lymphocytes. The expression of T lymphocyte subsets has value in judging the prognosis of ITP treated with GC (16). It's showed that CD4^+^/CD8^+^ ratio was an independent risk factor for GC ineffectiveness (cut-off: 1.02, AUC: 0.731, sensitivity: 53.4%, specificity: 87.5%).

The GC effective group showed higher C3 level than the ineffective group, while C4 levels did not differ significantly, consistent with the results of Shindo study (9). GC prevents the cytotoxic effect of complement on platelets through multiple pathways, such as inhibiting the synthesis of complement in the liver, increasing the expression of complement-regulated proteins, suppressing the production of pro-inflammatory cytokines, and weakening the cytotoxic effect. Cao's study suggested that the decrease of C3 was an independent influencing factor for the occurrence of chronic ITP in children with the AUC of 0.635 (30). In this study, the AUC was 0.731, the sensitivity was 56.9%, the specificity was 81.2%, and the cutoff value was 0.86 g/L.

The AUC for the above indicators to independently predict the ineffectiveness of GC treatment is all above 0.7, that is, the above indicators have predictive efficacy for the ineffectiveness of GC treatment and can be used as biological indicators to assist in the judgment of GC efficacy in children with ITP. However, none of the individual indicators achieved both high sensitivity and specificity simultaneously, suggesting limitations in their standalone predictive utility. Therefore, this study further conducted a combined test with the AUC of 0.919, and the sensitivity and specificity were 87.5% and 81.8%. The predictive value of combined detection is higher than that of the single detection.

Michael has suggested that a reduction in LYMPH may reflect a deficiency in CD4+ regulatory T cells (31). In this study, LYMPH levels were significantly lower and PLR values significantly higher in the IVIG ineffective group, indicating more pronounced immune dysregulation in IVIG ineffective group. Elevated PLR was an independent risk factor for IVIG failure in ITP children. The predictive cut-off value was 8.91%, the AUC was 0.769, the sensitivity and specificity were 54.5% and 87.3%. PLR had low sensitivity but good specificity in predicting ineffective IVIG treatment.

The occurrence of ITP is closely linked to humoral immunity, and the disorder of humoral immunity is more obvious in children with high expression of IgG. Previous studies have shown that decreased IgM is associated with complement activation and the occurrence of refractory ITP (32). In this study, the IgG level in the effective group was lower and the IgM level was higher than in the ineffective group. IVIG competitively inhibits Fc receptor-mediated antibody binding in the reticuloendothelial system of the spleen, down-regulates the production of autoantibodies by B lymphocytes, and accelerates antibody clearance in the circulation, thus playing an intervening role in the humoral immune response (33). It's showed that elevated IgG level was an independent risk factor, which was consistent with the results of previous studies (32). The cut-off value was 13.84 g/L, the AUC was 0.722, the sensitivity and specificity were 90.9% and 52.7%. Its specificity was lower than that of PLR, but the sensitivity was higher.

The combined analysis showed that the AUC of combined to predict the efficacy of IVIG treatment was 0.810, the sensitivity and specificity were 90.9% and 70.9%. Compared to single-index detection, the combined assessment provided more comprehensive and accurate information, demonstrating superior predictive performance and greater clinical relevance.

In conclusion, the increase of P-gp and PLR, CD4^+^/CD8^+^ ratio and decrease of C3 are independent risk factors for ineffective GC treatment in children with ITP, and the increase of PLR and IgG are independent risk factors for ineffective IVIG treatment in children with ITP. The above indicators have certain predictive value for the efficacy of first-line treatment in children with ITP. Moreover, the prediction efficiency of multi-index combination is better than that of single index. The early detection of these markers in children with ITP can predict their response to GC and IVIG based on the detection results, thus providing an important basis for the selection of clinical treatment options.For example, if the test results show that the child has a high risk of GC resistance and a high possibility of response to IVIG treatment, IVIG can be preferentially recommended as the first-line treatment to improve the treatment efficiency, reduce the occurrence of adverse drug reactions and ineffective medication, and achieve more targeted and personalized clinical decision-making. This study is a single-center and small-sample study, and the conclusion has certain limitations. In the future, it needs to be further verified through multi-center and large-sample studies to provide evidence support for the early assessment of the prognosis and outcome of first-line drug treatment in children with ITP.

## Data Availability

The original contributions presented in the study are included in the article/Supplementary Material, further inquiries can be directed to the corresponding authors.
